# Radiation dosimetry changes in radiotherapy treatment plans for adult patients arising from the selection of the CT image reconstruction kernel

**DOI:** 10.1259/bjro.20190023

**Published:** 2019-07-30

**Authors:** Anne T Davis, Sarah Muscat, Antony L. Palmer, David Buckle, James Earley, Matthew G.J. Williams, Andrew Nisbet

**Affiliations:** 1 Department of Physics, Faculty of Engineering and Physical Science, University of Surrey, UK; 2 Department of Medical Physics, Portsmouth Hospitals NHS Trust, Portsmouth, UK; 3 Department of Medical Physics, Royal Berkshire NHS Foundation Trust Reading, UK; 4 Department of Medical Physics, Royal Surrey County Hospital NHS Foundation Trust, Guildford, UK; 5 Department of Medical Physics, Velindre University NHS Trust, Cardiff, UK

## Abstract

**Objective:**

The reconstruction kernel used for a CT scan strongly influences the image quality. This work investigates the changes in Hounsfield units (HUs) which can arise when altering the image reconstruction kernel for planning CT images and the associated changes in dose in the radiotherapy treatment plan if the treatment planning system (TPS) is not re-calibrated.

**Methods:**

Head and neck, prostate and lung CT images from four centres were used. For a specific scan, the base image was acquired using the original reconstruction kernel (used when the TPS was calibrated) and the treatment plan produced. The treatment plan was applied to all images from the other reconstruction kernels. Differences in dose-volume metrics for the planning target volume (PTV) and organs at risk (OARs) were noted and HU differences between images measured for air, soft tissue and bone.

**Results:**

HU change in soft tissue had the greatest influence on dose change. When within ±20 HU for soft tissue and ±50 HU for bone and air the dose change in the PTV and OAR was within ±0.5% and ±1% respectively.

**Conclusions:**

When imaging parameters were changed, if HU change was within ±20 HU for soft tissue and ±50 HU for bone and air, the change in the PTV and OAR doses was below 1%.

**Advances in knowledge:**

The degree of dose change in the treatment plan with HU change is demonstrated for current TPS algorithms. This adds to the limited evidence base for recommendations on HU tolerances as a tool for radiotherapy CT protocol optimization.

## Introduction

The CT images used for radiotherapy treatment planning must be of good geometric fidelity and of sufficiently high image quality to allow accurate outlining of tumour volumes and organs at risk. The quality of a CT image is primarily dependent on the scan protocol settings.^1^ Parameters in all CT scan protocols should be set to ensure that optimal levels of image quality and imaging dose are delivered. It is usual practice for the protocols on a diagnostic CT scanner intended for imaging different clinical conditions or body regions to have different settings for the various scan parameters. Radiotherapy CT protocols should also be set to provide levels of contrast and spatial resolution which are appropriate for the particular body region imaged and size of patient.^2^ The task of accurately outlining the tumour and organs at risk on radiotherapy CT planning images is a demanding one and variability and inaccuracies are known to be a key source of uncertainty in the treatment planning process.^3–6^ The quality of radiotherapy CT images should support this process. The CT reconstruction kernel, which is selectable by the operator, is an important part of the image production process and can have a significant effect on the quality of the final CT image.^6–9^ Some CT scanners have many reconstruction kernels available for selection, although the kernels are usually developed with a specific imaging task in mind, such as imaging soft tissue or bony detail, or imaging in the head or body regions.^10,11^

The conversion of Hounsfield unit (HU) information in the CT image into attenuation information which can be used in the radiotherapy treatment planning system (TPS) requires a calibration curve. This is a plot of HU against relative electron density (RED) for a range of different density materials.^12^ Measurements to derive an HU-RED calibration curve are part of the commissioning process of a TPS. Some TPS systems allow the use of more than one curve, although it is not uncommon for centres to restrict the number used. This may be done to limit the burden of quality control testing on clinical settings and minimize the risk if the wrong calibration curve is inadvertently selected.^13^ The HU values obtained for the calibration curve depend predominantly on the design of the CT scanner, the CT scan parameters used and the shape, composition and positioning of the calibration phantom used.^14,15^ The scan parameters set when collecting HU information for the RED curve should match those which will be used when obtaining clinical planning scans.^14^ Reconstruction kernels can affect HU values.^6^ Radiotherapy guidance documents advise setting reconstruction kernels during the TPS commissioning phase.^7^ The degree of HU change for different reconstruction kernels on the various makes and models of CT scanners is only sparsely commented on in the scientific literature.^16,17^

Evidence from a recent UK wide survey indicates that many radiotherapy centres, for a specific scanner, often use the same, single reconstruction kernel irrespective of the region of the body being imaged.^18^ If consideration is to be given to adjusting the reconstruction kernels to improve the quality of the radiotherapy CT scans, the impact on the treatment planning process must be understood. The purpose of this work was to assess the degree to which HUs change when reconstruction kernels are altered, and to establish the corresponding change of dose calculated in the radiotherapy treatment plan. The change of dose within the TPS is known to be dependent on a number of factors including the planning algorithms, the treatment beam energy, the number of beams used, and the composition and thickness of the tissues in the beam.^12,19^ A previous literature review identified a lack of recent published work in this area, with no published papers related to current TPS algorithms or CT scanner reconstruction kernels and a lack of agreement on recommended tolerances between various standards and guidance documents.^20^ The conclusion from the review was that it may be appropriate to set tolerances of ±20 HU for soft tissue and of ±50 HU for the lung and bone if aiming to limit dose change in the treatment plan to within ±1% when compared with a base plan with no HU change. A very recent reference from IPEM quotes ±30 HU change in soft tissue to restrict dose change to ±1%, with ±2% dose change in lung and bone associated with HU change of ±50 and ±150 HU respectively.^13^ The published reference associated with those tolerances in the IPEM report is from 2005 and is relatively old. The setting of appropriate HU tolerances would allow, during scan protocol optimization work, the early discarding of any other scan protocol changes which cause HU change in excess of these tolerances. A clear need to test the proposed tolerances using data from modern TPS systems and CT scanners was identified. This work sets out to do that. Changes to clinical CT image quality arising from scan protocol changes have not been assessed as part of this study and will be investigated in the future. Previous work using an image quality phantom has demonstrated that the visibility of low contrast details and high contrast resolution can change significantly when the reconstruction kernel is changed, thus highlighting the importance of choosing the best reconstruction kernel to suit the imaging task.^21,22^

## methods and materials

The clinical CT scans chosen for this study were for patients requiring radiotherapy treatment for tumours in the head and neck, prostate and lung. The reason for selecting these three types of scan was to ensure that the plans assessed contained different volumes of bone, air and soft tissue. Breast plans were not included in this study since, although a large proportion of work for many centres, they are less complex in terms of treatment plan contouring and were not considered a particular focus for CT scan image quality improvement. Similarly palliative treatments were not included in high numbers within the sample as the treatment techniques are less demanding than for radical treatments. Four UK radiotherapy centres each using a different combination of TPS and CT scanner contributed data to the study, see Table 1. All CT images were acquired at 120 kV. The linear accelerator manufacturer and treatment beam energy at each centre was as follows: centres P and E were Varian (California, USA) with 6 MV for all tumour sites; centre M was Elekta (Stockholm, Sweden) with 6 MV for head and neck and 10 MV for prostate; centre R was Elekta with 6 MV. Altogether CT image data sets were used from 13 different patients (6 head and neck, 4 prostate and 3 lung). The study involved only external beam megavoltage photon treatment beams.

**Table 1.** The different treatment planning systems and CT scanners in use at the four centres in this study

Centre	Treatment planning system (software version)	Planning algorithms	CT scanner make and model	CT reconstruction kernel for baseline images (see Table 3)
P	Pinnacle (9.6) from Philips Healthcare (Best, Netherlands)	CCC; AC	Toshiba Medical Systems Ltd, now Canon Medical Systems (Tochigi Prefecture, Japan) Aquilion LB	FC13
E	Eclipse (11.0.31) from Varian Medical Systems (Calfornia, USA)	AAA	GE Healthcare (Chicago, USA) Lightspeed 16	Standard
M	Monaco (3.3.30) from Elekta (Stockholm, Sweden)	MC Photon	GE Healthcare (Chicago, USA) Lightspeed RT 16	Standard
R	Raystation (v 3.2) from Ray Search Laboratories (Stockholm, Sweden)	CC	Siemens Healthineers (Erlangen, Germany) Sensation Open	B31s+

AAA, Analytical Anisotropic Algorithm; AC, Adaptive Convolve; CC, Collapsed Cone; CCC, Collapsed Cone Convolution; MC, Monte Carlo.

The decision was made to use clinical images rather than an HU calibration phantom as such a phantom does not mimic the shape, size and composition of a patient across different body regions. HU values obtained in any CT image are in part determined by the patient/phantom shape, size and tissue adjacencies.^15^ The IAEA 2008 guidance on calibrating a TPS recommends the use of an anthropomorphic phantom containing appropriate materials mimicking clinical tissue types.^14^ An anthropomorphic phantom was not available. CT reconstruction kernels are intended for use on specific body regions and may behave differently when imaging an HU calibration phantom compared with a patient or anthropomorphic phantom. Similarly, since the dose change in a treatment plan resulting from HU changes will depend on the type and thickness of tissue that the treatment beams pass through, real clinical cases were deemed preferable to a non-anthropomorphic HU phantom.^20^

For a particular patient and tumour site, the CT image raw data was reconstructed using different CT reconstruction kernels so as to produce several sets of images related to that patient. The purpose of using different reconstruction kernels was to produce various degrees of HU change. One scan set was chosen and labelled as the baseline data set, a planning target volume (PTV) was defined and a treatment plan produced following the standard techniques routinely used in the radiotherapy centre where the images were acquired. Keeping the treatment monitor units (MUs) constant, the treatment plan was applied to the other CT data sets (those with different reconstruction kernels) for that patient. Across the different centres the PTV parameters recorded varied but the complete list of parameters were PTV D99%, D98%, D50%, D2% and mean dose to PTV. PTV D98%, for example, describes the dose received by 98% of the target volume. The doses to the organs at risk were also recorded. The OARs selected in each plan varied according to the exact position of the tumour site and the configuration of the treatment plan. Across all the plans the OARs reviewed are indicated in Table 2. For some OARs the parameter recorded was dose to a volume or mean dose and for others, the volume at a specified dose level.^23,24^ The chosen plan metrics were, in general, specified by the national clinical trial which each patient was enrolled on as part of their treatment or which had formed the basis for the clinical protocol in use. The priority of PTV or specific OAR was dependent on the clinical condition and the treatment objective for each plan. The dosimetric differences between the plans, relative to the baseline images, were assessed for both PTV and the OARs.

**Table 2.** The scan sets used indicating combination of anatomical site, CT reconstruction algorithm, TPS algorithm, treatment technique and prescription used

Centre	Scan set	CT reconstruction kernels	Anatomical site, patient size and gender	Treatment technique	Organs at risk	TPS algorithm	Dose grid resolution in three dimensions (cm)	Prescription dose (Gy) [number of fractions]
P	P1	Toshiba: FC13, FC23, FC41, FC44	Head and neck; superficial close to ear. Small female	IMRT; five fields: LAO25, LAO55, LPO95, LPO145, POST170	1 cc cord, parotid	CCC	0.25	55 [20]
P	P2	Toshiba: FC13, FC23, FC41, FC44	Head and neck; throat. Large male	VMAT; 2 arcs of 360°	1 cc cord, parotid	AC	0.25	Simultaneous boost 65.1 and 54 [23]
R	R1	Siemens: B31s, B10s, B30s, B60s	Head and neck. Large male	VMAT; 2 arcs of 360°	1 cc cord, parotid, 0.1 cc brainstem	CC	0.3	Simultaneous boost 66 and 54 [23]
E	E1	GE: Std, Soft, Edge, Detail, Bone, Bone plus	Head and neck. Medium male	Conformal, MLC defined; four fields	1 cc cord, parotid	AAA	0.25	30 [10]
M	M1	GE: Std, Soft, Edge, Detail, Bone, Bone Plus	Head and neck. Medium female	VMAT; 2 arcs of 360°	1 cc cord, 0.1 cc brainstem, parotid	MC	0.25	65.1 [23]
M	M2	GE: Std, Soft, Edge, Detail, Bone, Bone Plus	Head and neck – throat. Medium male	VMAT; 2 arcs of 360°	1 cc cord, 0.1 cc brainstem, parotid	MC	0.25	60 [23]
P	P3	Toshiba: FC13, FC23, FC41, FC44, FC03, FC08	Prostate. Medium male	VMAT; 150 to 210 degrees	Rectum, bladder	AC	0.25	Simultaneous boost 60 and 48 [20]
E	E2	GE: Std, Soft, Detail, Bone, Bone plus	Prostate. Medium Male	VMAT; two arcs	Rectum, bladder, bowel	AAA	0.25	78 [20]
M	M3	GE: Std, Soft, Detail, Bone, Bone Plus	Prostate. Medium Male	VMAT; 1 arc of 360°	Rectum, bladder, femoral head	MC	0.25	60[20]
M	M4	GE: Std, Soft, Detail, Bone, Bone Plus	Prostate. Medium Male	VMAT; 1 arc of 360°	Rectum, bladder, femoral head	MC	0.25	60 [20]
P	P4	Toshiba: FC13, FC41, FC44, FC23	Lung. Medium male	IMRT; five fields: Post180, LPO140, LPO100, LAO60, RAO340	1 cc cord, heart	CCC	0.2	55 [20]
P	P5	Toshiba: FC13, FC41, FC03, FC50, FC53	Lung. Medium male	IMRT; five fields: Post180, LPO140, LPO100, LAO60, RAO340	1 cc cord, heart	CCC	0.2	55 [20]
E	E3	GE: Std, Soft, Edge, Detail, Bone, Lung	Palliative Lung. Medium male	Parallel opposed fields	Carina	AAA	0.25	20 [5]

AAA, Analytical Anisotropic Algorithm; AC, Adaptive Convolve; CCC, Collapsed Cone Convolution; MC, Monte Carlo.

**Figure 1.**
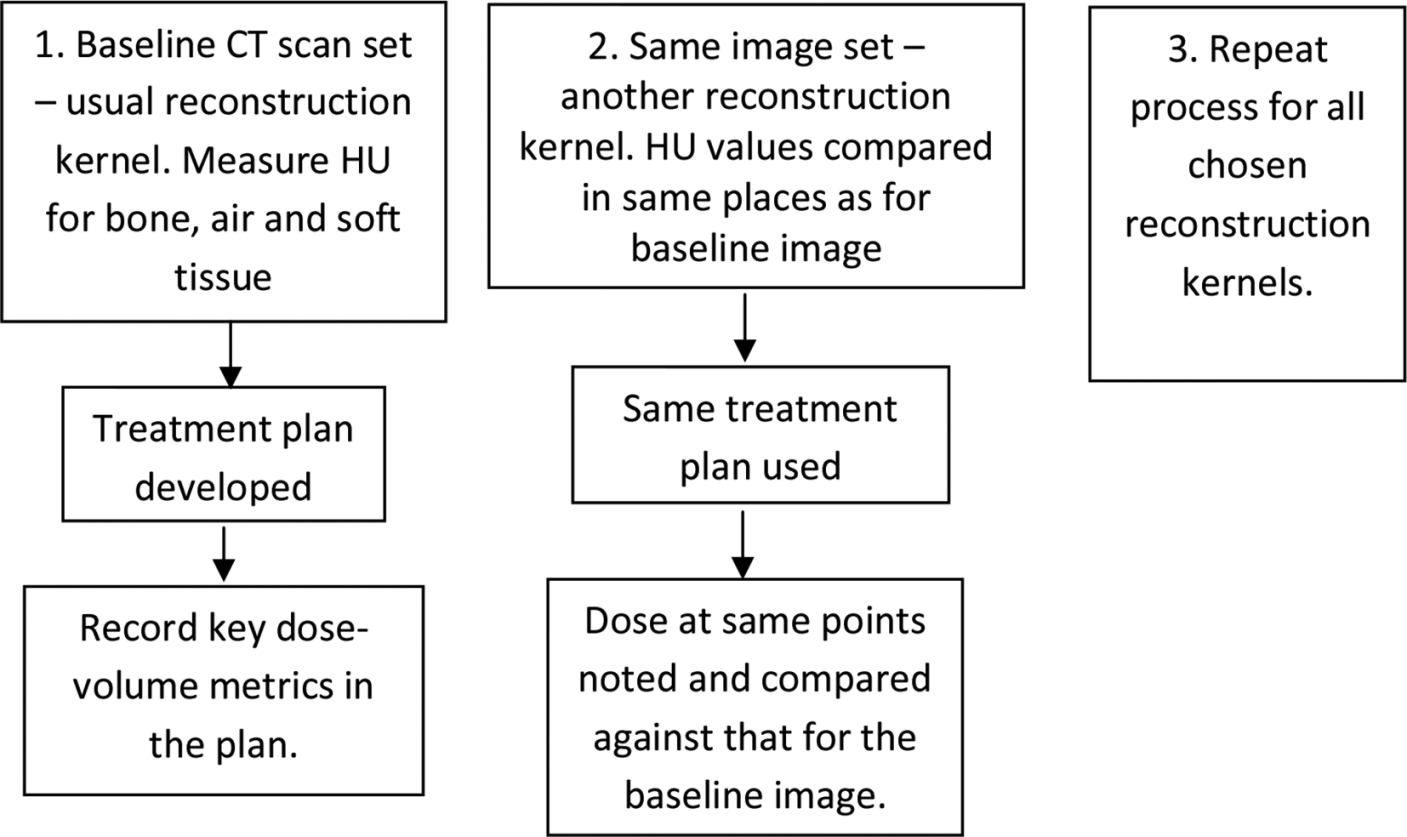
Flow diagram showing methodology used on a single scan set at one centre. HU,Hounsfield unit.

**Figure 2.**
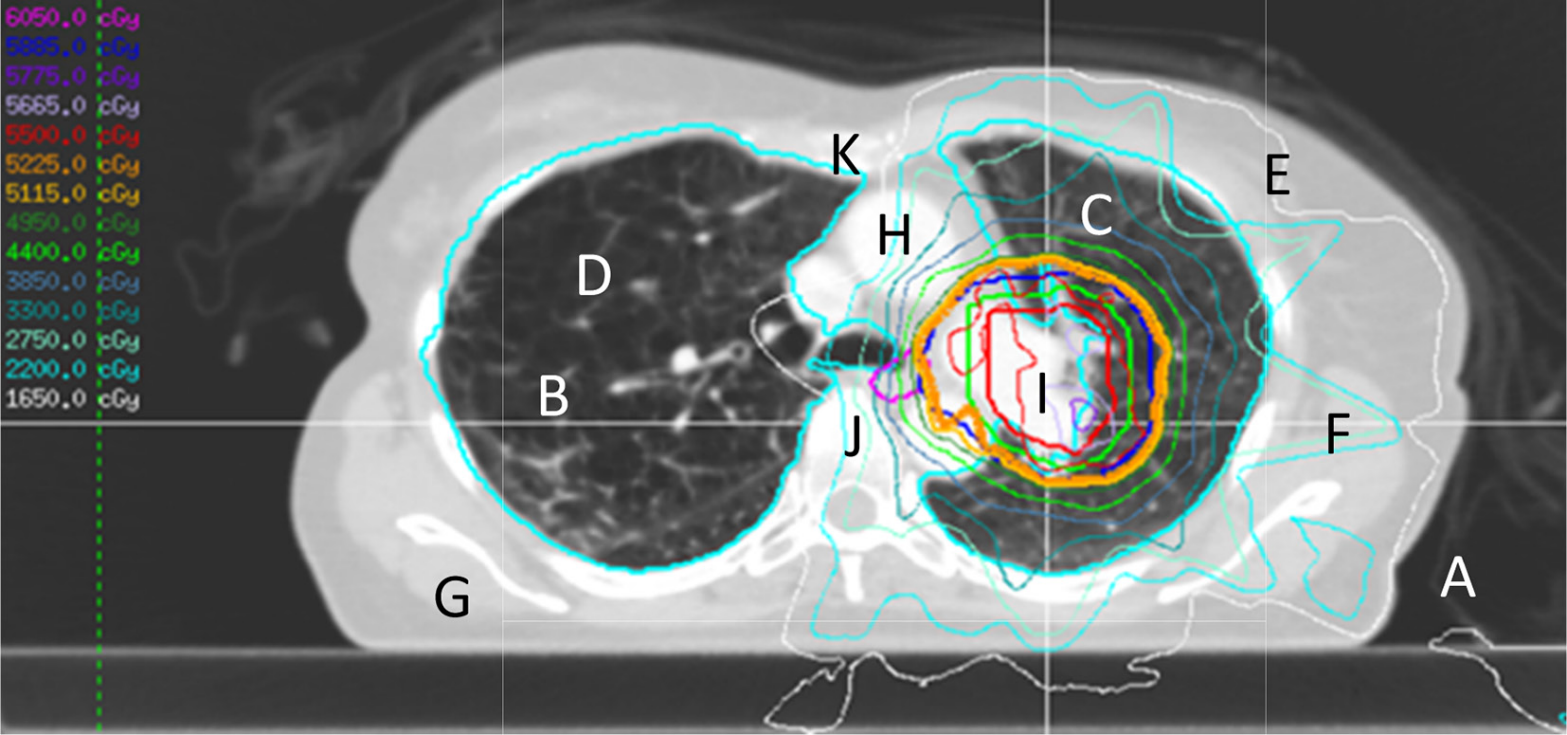
An example of one of the images showing the treatment plan and points where HU values were measured. HU,Hounsfield unit.

**Figure 3.**
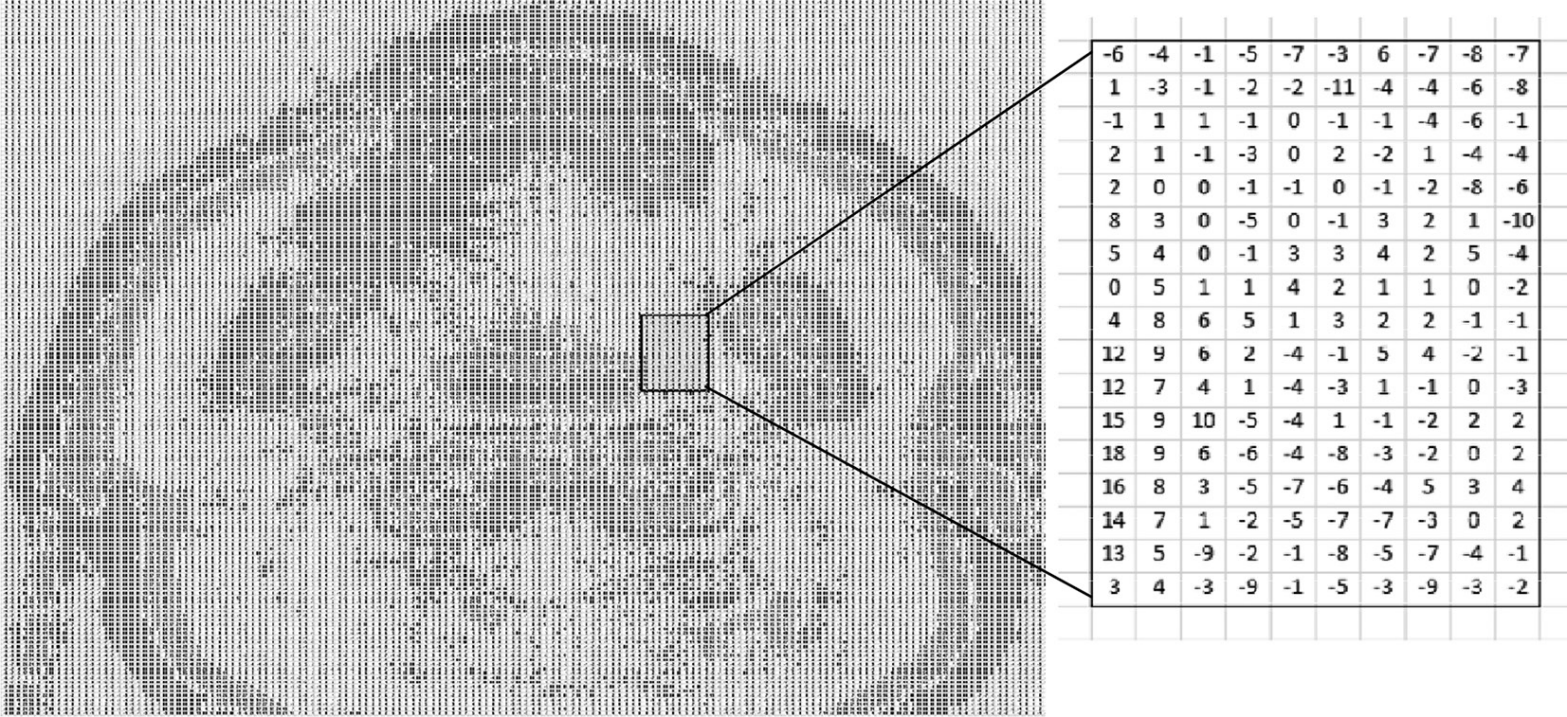
Example of the output of the Matlab code to subtract two images, showing CT number change per pixel

This process was repeated for each set of images. See Figure 1 for an overview of the measurement process. The baseline data set was acquired with scan protocol settings, including reconstruction kernel, which matched those which had been used to produce the RED to HU calibration curve in the TPS. An image at the isocentre was selected from within the set. Hounsfield units were measured for soft tissue, bone and air on the baseline plan, with a region of interest (ROI) sized and positioned to avoid measuring HU for more than one tissue type, see example in Figure 2. The co-ordinates of the ROIs were noted and the same positioning was used on each of the plans. Where possible, HU measurements were made on the CT scanner. In a few instances where HU was measured in the TPS a prior check was carried out to ensure there was an exact match of HU values for the CT scanner and the TPS. Finally, to validate the HU measurements, an alternative method of checking HU difference across the whole image was introduced. For the images which appeared to show the greatest HU change, software code developed in Matlab (Mathworks, Massachusetts) was used to subtract the base image from its paired image. This provided an HU image difference map (see example in Figure 3) and gave confidence that the results obtained using ROIs were representative of the image as a whole.

From 13 sets of images, see Table 2, there were 13 baseline images and 53 other images. This enabled 53 separate sets of comparisons to be made of HU change for soft tissue, bone and air against the dose change in the treatment plan. Since the purpose of changing the reconstruction kernel was to force an HU change in the CT image, a range of different kernels were selected depending on what was available on the CT scanner. In some instances this included using kernels intended for body regions which did not match the anatomical site. This reflects current practice in many UK radiotherapy centres where CT scanners often image all head and body sites with a single kernel intended for body imaging.^18^ The intended clinical use of the various kernels selected is indicated in Table 3. One further set of measurements (using scan set P2, see Table 2) were made to investigate the impact of using a flattening-filter-free (FFF) beam on a Varian linear accelerator. A treatment plan using 6 MV FFF was developed, applied to scan set P2 (Table 2) and the HU and dosimetry differences compared.

**Table 3.** CT manufacturer’s intended use for the CT reconstruction kernels used in this work^25–27^

Toshiba Medical Systems Ltd	FC03	Abdomen—with BHC
	FC08	Abdomen—with BHC; increased contrast
	FC13	Body—without BHC
	FC23	Head—with BHC; fine grain size
	FC41, FC44	Head—without beam hardening correction. (FC44 sharper than FC41).
	FC50, FC53	Lung (FC53 sharper than FC50)
Siemens Healthineers	B10s	All these intended for any region in the trunk. The higher number indicates a sharper kernel. 15 body kernels are available on scanner ranging from B10s to B80s. Separate kernels are available for head imaging.
	B30s	
	B31s	
	B60s	
GE Healthcare	Soft	Tissues with similar densities but not for un-enhanced scans
	Standard	Routine examinations, *e.g.* chest, abdomen and pelvis
	Detail	Where bone edges are important
	Bone	High resolution and sharp bone detail
	Bone plus	For sub mm head imaging
	Edge	Small bone imaging in the head and high resolution scans.
	Lung	For imaging lungs

BHC, beam hardening correction.

## Results

**Figure 4.**
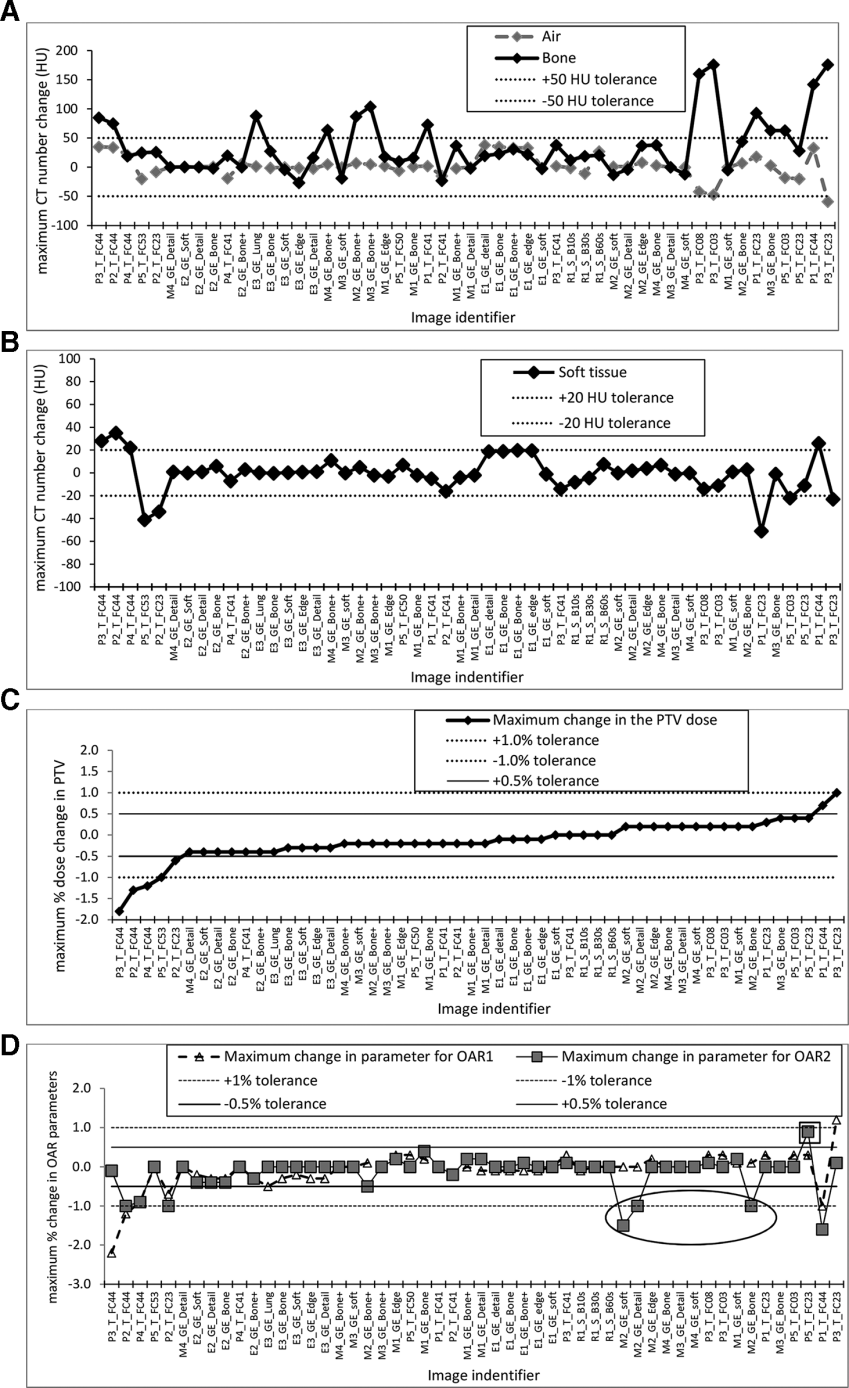
For each image, when compared to a baseline image, the changes in (a) HU for air and bone, (b) HU for soft tissue with (c) the corresponding maximum change in the PTV dose and (d) the maximum change in the dose parameter for two OAR in the treatment plan. Data points are sorted in order of ascending values of dose change in the PTV. HU, Hounsfield unit; OARs, organs at risk; PTV, planning target volume.

For each image, when compared against a baseline image, the values of maximum HU change for air, bone and soft tissue were plotted alongside the maximum change in PTV dose quantities and maximum change in either volume at specified dose or in dose level for two OARs. The data points were plotted in order of increasing dose change in the PTV. See Figure 4 (a-d). The tolerances used, ±50 HU for air and bone and ±20 HU for soft tissue, when compared against the values in the baseline images, are those proposed in the introduction as corresponding to a dose change of within ±1% and are based on a detailed literature review.^20^ After reviewing results, a tighter tolerance of ±0.5% was also added to the graphs for PTV and OAR dose change, Figure 4c and d.

The degree of HU change depended greatly on the selected reconstruction kernel with some resulting in little or no change (less than 5 HU for all tissue types), whilst others kernels produced large changes (over 30 HU for soft tissue and more than 100 HU for bone). The largest HU changes were seen for some of the images created using reconstruction kernels on the GE (Chicago, USA) and Toshiba (now Canon, Tochigi Prefecture, Japan) CT scanners. The images reconstructed using the Siemens reconstruction B (body) kernels show HU changes close to zero. This is in line with other published work.^16,17,21^ The results for the data set where a flattening filter free 6 MV beam was used gave results which were very similar to the standard 6 MV beam. The degree of HU change can be compared with the level expected from day-to-day variation of CT scanner performance as seen in quality control (QC) testing, typically less than ±5 HU for water, analogous to soft tissue, and less than ±10 HU for bone.^28^ The HU difference arising from a change in the reconstruction kernel is, in some cases, considerably greater than these quality control tolerances.

Changes in the soft tissue HU had the largest impact on any change in dose with all seven points where PTV dose change is greatest (exceeding ±0.5%) corresponding to soft tissue HU changes which exceed the ±20 HU tolerance, see Figure 4(b) , and (c). For the OARs, the parameter change also generally exceeded ±0.5% where the ±20 HU tolerance was exceeded for soft tissue HU change. For three images, OAR parameters changed by more than ±0.5% without a significant HU change in soft tissue HU. All these points, marked with an ellipse in Figure 4(d), correspond to a dose to the brain stem for a volume of 0.1cc. This volume is relatively small and therefore more subject to change. One further point in Figure 4(d), identified with a square box, shows one other OAR where the parameter change is close to the +1% level. This dose difference (11.6 *vs* 11.5 Gy to the heart which is 0.9%) is within the quoted precision of 0.1 Gy for the recorded dose.

The HU changes for air exceeded the proposed ±50 HU tolerance in only one case and in that instance the dose change in the PTV also exceeded the ±1% tolerance. The ±50 HU tolerance for bone is exceeded in 14 cases out of 53 (26%) but the ±1% dose change is only exceeded in four cases. This is in keeping with other work which suggests that it may be acceptable for bone HU to change by more than ±50 HU without adversely impacting on the treatment plan dosimetry.^29–31^ There is one point in Figure 4(b) where soft tissue HU exceeds the ±20 HU tolerance but maximum dose change in the treatment plan is minimal at −0.1%. The specific results for this point showed the HU change for bone to be positive and the change for soft tissue to be negative; indicating that changes in HU for different tissue types can counteract each other resulting, in this case, in no significant dose change in the plan.

## Discussion

This work has assessed four common radiotherapy TPSs employing four modern algorithms, with head and neck, prostate and lung images for 19 CT reconstruction kernels from three radiotherapy CT scanner models. This provides an update to the literature to include models of radiotherapy CT scanners in use today, using a range of different body regions and treatment techniques, and looking specifically at how HU change arising from different reconstruction kernels corresponds to dose change in the treatment plan. The results show that if HU changes caused by modification of the CT scan parameters are kept to within ±20 HU for soft tissue and ±50 HU for air and bone, any dose changes in the treatment plan for both PTV and OAR parameters will be within ±1% compared to a base plan, and in all cases within ±0.5% for the change in the PTV. Results from the four TPS and different planning algorithms are spread through the data set with no evidence of clustering. There is therefore no suggestion of the treatment planning algorithm used having a strong influence on these results. The three examination types, head and neck, prostate and lung are also mixed up across the data set.

These results will be of use when optimizing CT scan protocols for the purpose of image quality improvement or dose reduction as initial measurements of HU change can be made on the CT scanner. Likely changes to HU arising from any other variables, such as routine day to day variability in scanner performance, should also be allowed for.^13^ Treatment plans produced for the images using the selected reconstruction kernels can then be assessed in the TPS and a decision made as to whether the original TPS calibration curve can be used.

Limitations of this work include the fact that the treatment beam energy used for the majority of the plans was 6 MV, with only two using higher energy treatment beams. The evidence in the literature, however, is that higher beam energies result in a lower level of dose change in the treatment plan for a given change in HU.^32^ It is notable that the results when using a FFF 6 MV treatment beam are similar to those using a standard 6 MV beam despite the reduction in beam quality which results from use of FFF on a Varian treatment machine. The study covers only external beam megavoltage treatments. No inclusion has been made of the effect of HU change in treatment plans where tomotherapy, particle therapy, CyberKnife (California, USA), electron beams, stereotactic or small field treatment techniques are used. Those considerations are outside of the scope of this investigation.

In addition, the patient cohort selected was consistent with a standard range of adult patient sizes but did not include a comprehensive range of patient sizes including paediatrics and bariatric patients. Any centre exploring the impact of CT reconstruction kernel change should ensure a representative sample of the different patient sizes treated at that centre is included in their investigative work. Centres treating paediatric patients should take care to include a representative range of size/age ranges in their investigative work. Treatment plans for large patients in particular and those where tumours are deep and treatment beam paths pass through increased tissue volumes should be examined when changes in reconstruction kernel are considered as published literature indicates that for these conditions the dose change may be greatest for a given HU change.^30,33^ Treatment sites included in this study were limited to only three (head and neck, pelvis and lung) but the impact of CT scan protocol changes should be investigated for all sites treated.

It should also be noted that changing the CT reconstruction kernel may change the position of the visible edge of an organ in the image due to the degree of sharpness or blurring resulting from the reconstruction kernel.^34,35^ During measurement of HUs in this work care was taken to position regions-of-interest away from the boundaries between different tissue types to avoid this having an impact on measured HU values. Detailed checking of the impact of this effect should form part of the comprehensive assessment of image quality changes resulting from the use of different image reconstruction processes and parameters. Not all CT scanner types, and their associated reconstruction kernels, have been included in this study and the use of the newer iterative reconstruction algorithms and their effect on HU or treatment planning dose is not within the scope of this work.

As an aside, it is interesting to consider the impact of the use of iodine based contrast agents (CA) in radiotherapy CT scanning. When administered, CAs perfuse areas in and around vascular structures and help to improve visibility and subsequent delineation of the tumour volume and organs at risk. The presence of the CA increases the HU values for the tissues reached during the CT scan. The degree of HU increase depends on the concentration of the CA; higher concentrations increase the HU values.^36^ The subsequent dose difference in the TPS depends upon the diameter of the tissue/lesion containing the CA, the extent of the HU increase, the treatment beam energy, the number of incident beams and the TPS algorithm.^36–38^ Some studies have seen CA cause increases of several hundred HU, primarily in areas of high blood flow. Corresponding changes in treatment planning dose do depend on the TPS algorithm and treatment regime but for the lung and heart regions dose change was reported as approximately 1– to 3% related to HU changes between approximately 100 and 450.^36–38^

Finally, it is appropriate to briefly mention the degree of inaccuracy discussed here for the TPS HU to treatment dose conversion process within the context of other uncertainties which exist within the radiotherapy process. The treatment process is complex and contains many steps, each of which introduces uncertainties with differing degrees of magnitude. Two examples of these are the day–day variability of a linear accelerator output which is in the region of ±2% and the differences between measured dose and those calculated by different TPS algorithms which are estimated as being within 3.5% for complex algorithms and up to 20% for simple ones.^39,40^ Uncertainties at all stages of radiotherapy are discussed in detail by others.^41,42^ For the part of the process related to treatment planning, the IAEA and IPEM define acceptable criteria as being between 2 and 5%, depending on the specific treatments and geometries.^12,43^ It is well known that variability in clinical contouring introduces the largest uncertainty in the treatment process.^39,43^ Image quality is one of a number of factors contributing to this.^20^ All attempts to optimize CT image quality with a view to increasing the visibility of organ boundaries and support optimal contouring are worthwhile. Whilst separate TPS calibration curves related to differing scanning protocols would be preferable, it is accepted that this may not be possible or desirable due to other considerations as discussed in "Introduction." In that instance a small difference in TPS calibration accuracy may be more than offset by improvement in clinical image quality resulting from changes to CT scan parameters.

## Conclusion

The production of good quality CT images is an important aspect of the radiotherapy treatment planning process where tumour and OARs are to be outlined. Site-specific CT scan protocols should contain parameters which produce optimal imaging for each body region and in line with clinical requirements.^2^ Adjustments of scan protocol settings to improve image quality and optimize imaging dose may include consideration of changing the image reconstruction kernel.^6^ Data from the 2018 UK audit of CT patient doses which collected scan protocol information showed that more than half (30 of 53) of the centres that contributed data were using a single reconstruction kernel for lung, brain, prostate and head and neck imaging. This suggests that there is scope on some scanners to improve image quality through changes to the reconstruction kernels. With careful selection, changing the reconstruction kernel used to produce radiotherapy CT images can result in only a small change in the dose in the treatment plan. Yet, it is well known that the reconstruction kernel will influence the quality of the final image^6,8,44,45^ Any changes to scan protocols should consider whether the optimum reconstruction kernel is in use. Further work to assess how image quality can be improved for CT images outlined both by clinicians and auto-contouring systems is therefore justified.

This work confirms that, when compared to the baseline image used to produce the TPS calibration curve, provided any change in CT number in a new image is within ±20 HU for soft tissue and ±50 HU for bone, the dose change in the treatment plan for the PTV and the OARs will be within ±1% and often within ±0.5% when the TPS calibration curve remains unchanged. Whilst all radiotherapy centres should undertake their own checks using centre specific combinations of treatment techniques, equipment, patient types and cancer sites before implementing any changes these tolerances may be used as a guide when making changes to the CT scan protocol settings for the purpose of optimization.
